# Electronic Structure Regulated Nickel-Cobalt Bimetal Phosphide Nanoneedles for Efficient Overall Water Splitting

**DOI:** 10.3390/molecules29030657

**Published:** 2024-01-31

**Authors:** Heyang Xu, Xilin She, Haolin Li, Chuanhui Wang, Shuai Chen, Lipeng Diao, Ping Lu, Longwei Li, Liwen Tan, Jin Sun, Yihui Zou

**Affiliations:** 1 State Key Laboratory of Bio-Fibers and Eco-Textiles, Shandong Collaborative Innovation Center of Marine Biobased Fibers and Ecological Textiles, Institute of Marine Biobased Materials, School of Environmental Science and Engineering, Qingdao University, Qingdao 266071, China; xyc1491@163.com (H.X.); xlshe@qdu.edu.cn (X.S.); lihaolin19950223@163.com (H.L.); wch2022020768@163.com (C.W.); luping@qdu.edu.cn (P.L.); llw10172023@163.com (L.L.); 2 State Key Laboratory of Coal Conversion, Institute of Coal Chemistry, Chinese Academy of Sciences, Taiyuan 030001, China; chenshuai@sxicc.ac.cn; 3 Qingdao Hanxing New Materials Co., Ltd., Qingdao 266109, China; diaolipeng@qdhanxing.com; 4 School of Material Science and Engineering, State Key Laboratory of Bio-Fibers and Eco-Textiles, Qingdao University, Qingdao 266071, China

**Keywords:** Ni_x_Co_2−x_P nanoneedles, electronic structure regulation, hydrogen evolution reaction (HER), oxygen evolution reaction (OER), water splitting

## Abstract

Transition metal phosphides (TMPs) have been widely studied for water decomposition for their monocatalytic property for anodic or cathodic reactions. However, their bifunctional catalytic activity still remains a major challenge. Herein, hexagonal nickel-cobalt bimetallic phosphide nanoneedles with 1–3 μm length and 15–30 nm diameter supported on NF (Ni_x_Co_2−x_P NDs/NF) with adjusted electron structure have been successfully prepared. The overall alkaline water electrolyzer composed of the optimal anode (Ni_0.67_Co_1.33_P NDs/NF) and cathode (Ni_1.01_Co_0.99_P NDs/NF) provide 100 mA cm^−2^ at 1.62 V. Gibbs Free Energy for reaction paths proves that the active site in the hydrogen evolution reaction (HER) is Ni and the oxygen evolution reaction (OER) is Co in Ni_x_Co_2−x_P, respectively. In the HER process, Co-doping can result in an apparent accumulation of charge around Ni active sites in favor of promoting HER activity of Ni sites, and ΔG_H*_ of 0.19 eV is achieved. In the OER process, the abundant electron transfer around Co-active sites results in the excellent ability to adsorb and desorb *O and *OOH intermediates and an effectively reduced ∆G_RDS_ of 0.37 eV. This research explains the regulation of electronic structure change on the active sites of bimetallic materials and provides an effective way to design a stable and effective electrocatalytic decomposition of alkaline water.

## 1. Introduction

Hydrogen, as a clean and sustainable new energy, has become the most potential substitute for non-renewable resources due to its high energy density [1,2,3,4]. Among various hydrogen production methods, electrochemical decomposition of water is an efficient and high-purity hydrogen production method, which can effectively reduce environmental pollution in the energy production process [5,6,7]. This process is two half-reactions composed of the hydrogen evolution reaction (HER) and oxygen evolution reaction (OER), and both of them require noble metals (Pt-based or Ru-based) to achieve excellent electrochemical performance [8,9,10,11]. However, their large-scale application is hindered by their scarcity and expensive cost. Therefore, it is imperative to develop an efficient and inexpensive electrocatalyst for the widespread commercialization of electrochemical water splitting.

Among the transition metal-based electrocatalysts, transition metal phosphides (TMPs) have been widely studied due to their abundant and inexpensive raw materials and hydrogenase-like structure, which could effectively reduce the cost of precious metal catalysts [12,13,14,15,16]. As a result, the HER and OER catalytic properties of TMPs have been thoroughly investigated, such as NiCoP nanofibers [17], Ni_2_P-CoP@NC [18], NiP_x_ nanospheres [19], hollow urchin-like FeP [20], and carbon encapsulating MoP [21]. Among the common transition metal phosphides with better activity, cobalt-based phosphides exhibit better OER activity, while in HER, nickel-based phosphides exhibit better performance. However, the dual functionality of these catalysts is still a challenge for practical application. Electronic structure regulation by doping heterogeneous elements is a beneficial method to enhance the electrocatalytic performance of nickel-based or cobalt-based phosphides [22,23,24,25,26]. For instance, W, Fe-doped CoP nanosheets exhibit significant improvement in OER performance (η_10_ = 290 mV) owing to the adjusted electronic structure by the incorporation of duplex metal, while its HER performance still has a lot to improve (η_10_ = 130 mV) due to the higher H* desorption energy barrier [27]. Jiang and co-workers reported that O-incorporated NiMoP nanotubes could provide high activity for HER performance (η_10_ = 54 mV), which benefits from the modulated electronic structure [28]. Nevertheless, its OER performance is still unsatisfactory, requiring 520 mV to reach 10 mA∙cm^−2^. This may be due to the absence of an active site for OER reaction intermediates [29]. Considering that Co^2+^ has suitable adsorption and resolution energy for intermediate O* of OER, and Ni^III^ could optimize H* adsorption kinetics to enhance alkaline HER performance [30,31], modulating the electronic structure of Co and Ni simultaneously will be an effective strategy to improve the bifunctional activity.

Herein, we successfully fabricated urchin-like NiCoP nanoneedles grown on nickel foam (denoted as Ni_x_Co_2−x_P NDs/NF). Benefiting from the optimized electronic structure via changing Ni/Co ratios, the Ni_0.99_Co_1.01_P NDs/NF electrode exhibits a low overpotential of 63 mV at 10 mA cm^−2^ for HER, while the overpotential of Ni_0.67_Co_1.33_P NDs/NF electrode is as low as 298 mV to attain a current density of 100 mA cm^−2^ in the OER process. Furthermore, the Ni_x_Co_2−x_P NDs/NF electrode exhibits outstanding overall water splitting performance (100 mA cm^−2^ at 1.62 V). In addition, DFT calculations were used to investigate electronic structure change on the active sites of bimetallic materials and provided theoretical direction to design a stable and effective electrocatalytic decomposition of alkaline water.

## 2. Results

### 2.1. Preparation and Structure Characterization of Ni_x_Co_2−x_P NDs/NF

**Figure 1:**
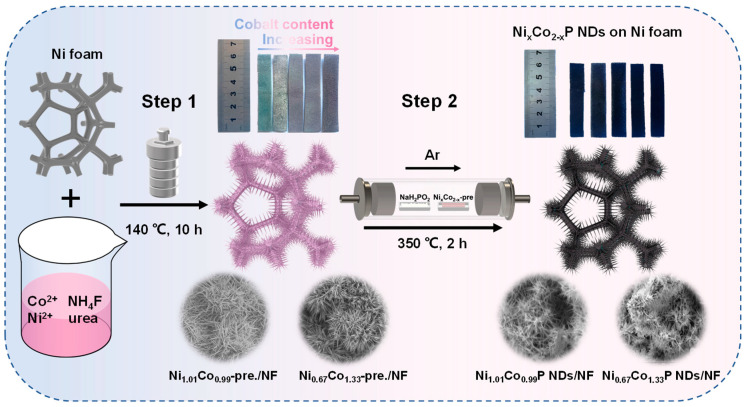
Schematic illustration of the preparation of Ni_x_Co_2−x_P NDs/NF supported on Ni foam.

The preparation procedure of Ni_x_Co_2−x_P NDs/NF is shown in Figure 1. In step 1, an appropriate amount of NH_4_F and urea interact with cobalt/nickel salts to form needle-like precursors by rapid deposition. In order to achieve the optimum conditions, the hydrothermal reaction parameters of Ni_x_Co_2−x_-pre. were gradually probed by adjusting the Ni/Co ratio. As the amount of Co doping increased, the color of Ni_x_Co_2−x_-pre. on NF shifted from blue to pink progressively. In step 2, the subsequent phosphatization process (2) generated a sea urchin-like nanoneedle structure through the reaction of the needle-like precursor with the decomposition product *PH_3_* of *NaH_2_PO_2_* (1) [32].

$$2NaH_{2}PO_{2}\cdot H_{2}O\to NaHPO_{4}\left(s\right)+H_{2}O\left(g\right)+PH_{3}\left(g\right)$$

$$M^{2+}+{PH}_{3}(g)\to M_{m}P_{n}\left(M^{2+}={Co}^{2+},{Ni}^{2+}\right)$$

Ni_x_Co_2−x_-pre. converted into Ni_x_Co_2−x_P with heat treatment under the protection of Ar atmosphere. It can be seen from the digital picture in Figure 1 that the color of NF turns black after low-temperature phosphorization.

**Figure 2:**
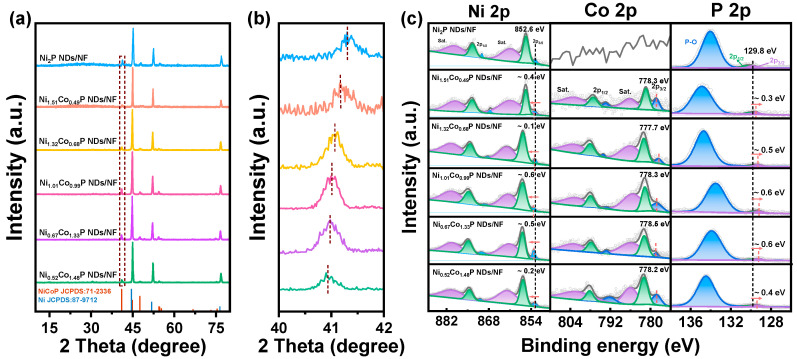
(**a**) XRD patterns of the Ni_x_Co_2−x_P NDs/NF with various Ni/Co ratios; (**b**) XRD results at the (111) peak in the marked region of (**a**); (**c**) XPS spectra of Ni_2_P ND/NF and Ni_x_Co_2−x_P NDs/NF: Ni 2p, Co 2p, and P 2p regions.

XRD was used to determine the phases of the prepared materials. To eliminate the high peak blockage of NF, the precursor powders stripped from NF via hydrothermal treatment were collected for XRD measurement (Figure S1). It can be discerned that the diffraction peaks of the precursor correspond to Ni(OH)_2_ (PDF #14-0017) and Co(CO_3_)_0.5_OH∙0.11H_2_O (PDF #48-0083), respectively [33]. As the XRD spectrum of Figure 2a depicts, the catalysts, after phosphating with different Ni/Co ratios, are all the same as the crystal structure of hexagonal Ni_2_P (PDF #71-2336), indicating that Co has been successfully doped into the Ni_2_P crystal [34]. The cell parameters are a = 5.834 Å, b = 5.834 Å, c = 3.351 Å; V = 98.8 Å^3^; space group P-62m (189). To determine the precise amount of Co doping in Ni_x_Co_2−x_P NDs, we conducted ICP–AES analysis. From Table S1, it can be concluded that the five samples present the Ni/Co atomic ratios of 1/0.326, 1/0.515, 1/0.973, 1/1.996, 1/2.845, which correspond to Ni_1.51_Co_0.49_P, Ni_1.32_Co_0.68_P, Ni_1.01_Co_0.99_P, Ni_0.67_Co_1.33_P, and Ni_0.52_Co_1.48_P, respectively. The peaks at 44.5°, 51.9°, and 76.3° are the fingerprint peaks of Ni (PDF #87-9712) [34]. It is worth noting that there is a red shift in major peaks via increasing fractions of Co (Figure 2b). That may be due to the substitution of Ni (124.6 pm) by Co (125.3 pm) with a higher atomic radius [35].

XPS was further used to characterize the chemical states of different elements in the Ni_x_Co_2−x_P ND/NF. In Figure S2, the co-existence of Ni, Co, and P elements has been observed in the Ni_x_Co_2−x_P NDs/NF, while Co is absent in the survey of Ni_2_P NDs/NF. As shown in Figure 2c and Table S2, high-resolution spectra of Ni 2p and Co 2p for Ni_x_Co_2−x_P NDs/NF exhibit two regions and one region for P 2p. For typical samples of Ni_1.01_Co_0.99_P NDs/NF and Ni_0.67_Co_1.33_P NDs/NF, there are four fitted peaks at ~853.2 eV (2p_1/2_), ~869.6 eV (2p_3/2_), ~856.7 eV (2p_1/2_), and ~875.0 eV (2p_3/2_), respectively, which are related to Ni–P species and oxidized Ni species. The peaks at ~861.2 and ~879.6 eV can be allocated to the satellite peaks of Ni 2p [36]. In Co 2p spectra of Ni_1.01_Co_0.99_P NDs/NF and Ni_0.67_Co_1.33_P NDs/NF, the peaks at 778.3 and 778.5 eV can be fitted into Co–P, while the peaks at ~782.0/~797.9 eV and ~786.1/~802.2 eV can be recognized as the oxidized Co species and satellite peaks, respectively [37]. There are characteristic peaks of phosphate and phosphide in P 2p narrow scan spectrum. The peak at the lower binding energy of ~129.5 eV could be assigned to P 2p_1/2_ and P 2p_3/2_ of phosphide, respectively, whereas the peaks at ~132 eV could contribute to the P–O originated from the oxidation after exposure to the air [38]. The addition of Co induces a red shift in P 2p3/2 and a blue shift in Ni 2p3/2, indicating an efficient electronic modulation. The change in the Ni/Co ratio is the key reason for the binding energy shift. The electron structure of the Ni position on Ni_1.01_Co_0.99_P NDs/NF becomes the most negative, and the Co position on Ni_0.67_Co_1.33_P NDs/NF becomes the most positive, which should be highly beneficial to accelerate catalytic reactions [39].

**Figure 3:**
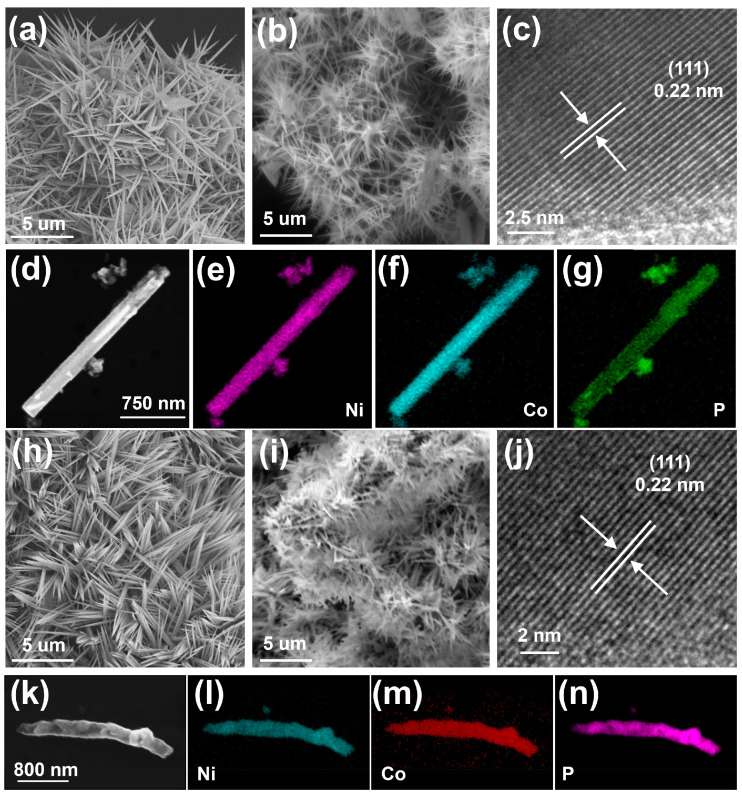
SEM images of (**a**) Ni_1.01_Co_0.99_-pre.; (**b**) Ni_1.01_Co_0.99_P NDs/NF; TEM images of (**c**) Ni_1.01_Co_0.99_P NDs; EDX element mapping images of (**d**–**g**) Ni_1.01_Co_0.99_P NDs; SEM images of (**h**) Ni_0.67_Co_1.33_-pre.; (**i**) Ni_0.67_Co_1.33_P NDs/NF; TEM images of (**j**) Ni_0.67_Co_1.33_P NDs; EDX element mapping images of (**k**–**n**) Ni_0.67_Co_1.33_P NDs.

The morphology of Ni_x_Co_2−x_-pre. and Ni_x_Co_2−x_P NDs/NF were characterized by SEM, and TEM was used to analyze the structure of Ni_x_Co_2−x_P NDs. Two samples with excellent electrochemical performance were selected as representative samples. The SEM images of Ni_1.01_Co_0.99_-pre. and Ni_0.67_Co_1.33_-pre. Figure 3a,d show the morphology of a sea urchin with a uniform needle shape. The Ni_1.01_Co_0.99_-pre. and Ni_0.67_Co_1.33_-pre. have an average length of approximately 2–4 μm and a diameter of 15–30 nm. In Figure 3b,e, the needle structures of Ni_1.01_Co_0.99_P and Ni_0.67_Co_1.33_P still exist; however, their surface becomes visibly rough and short in length (1–3 μm). This may be ascribed to the loss of the bonding water during the phosphating process. The one-dimensional needle shape could provide rapid transport of electrons, ions, and products of the HER and OER process and improve the contact area, which is beneficial for improving electrochemical performance [32]. HRTEM images of Ni_1.01_Co_0.99_P NDs and Ni_0.67_Co_1.33_P NDs are shown in Figure 3c,j, respectively. The same spacing of 0.220 nm was discovered to be consistent with the interplanar spacing of (111) plane for Ni_x_Co_2−x_P [40].

### 2.2. Electrocatalytic Activity for HER and OER

**Figure 4:**
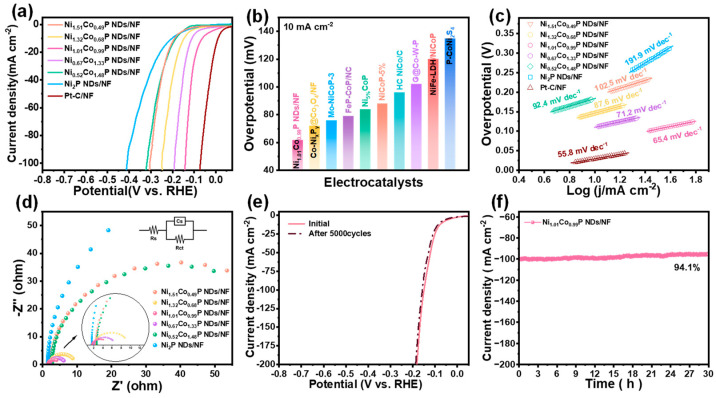
Electrocatalytic performance of Ni_x_Co_2−x_P NDs/NF for HER with different Ni/Co ratios in 1 M KOH: (**a**) iR-corrected LSV profiles; (**b**) overpotential comparison at 10 mA cm^−2^ with recently reported electrocatalysts; (**c**) Tafel plots; (**d**) EIS spectra (inset shows the corresponding equivalent impedance circuit diagram); (**e**) comparison of LSV profiles of Ni_1.01_Co_0.99_P NDs/NF before and after 5000 CV cycles; (**f**) time-dependent current density curve of Ni_1.01_Co_0.99_P NDs/NF under static potential of −1.08 V for 30 h.

The electrocatalytic performance of all as-prepared catalysts toward HER was evaluated by linear sweep voltammetry in 1.0 M KOH solution at 5 mV∙s^−1^. As shown in Figure 4a, Ni_1.01_Co_0.99_P NDs/NF exhibit preferable HER performance compared with that of Ni_1.51_Co_0.49_P NDs/NF, Ni_1.32_Co_0.68_P NDs/NF, Ni_0.67_Co_1.33_P NDs/NF, Ni_0.52_Co_1.48_P NDs/NF, and Ni_1.01_Co_0.99_P NDs/NF. In addition, to achieve the current density of 10 mA∙cm^−2^, the Ni_1.01_Co_0.99_P NDs/NF only require an overpotential of 62 mV, which is apparently lower than that of Ni_1.51_Co_0.49_P NDs/NF (190 mV), Ni_1.32_Co_0.68_P NDs/NF (190 mV), Ni_0.67_Co_1.33_P NDs/NF (110 mV), Ni_0.52_Co_1.48_P NDs/NF (184 mV), Ni_2_P NDs/NF (202 mV), and previously reported TMPs catalysts (Figure 4b and Table S3) [41,42,43,44,45,46,47,48,49]. The experiment results show that Co doping could effectively improve the HER activity of Ni_2_P. The most optimal Ni/Co atom ratio is 1/1. Moreover, the better HER activity of Ni_1.01_Co_0.99_P NDs/NF compared with that of Ni_1.01_Co_0.99_-pre. indicates the importance of the phosphorization in the hybrid electrode. Furthermore, to probe into the consequence of Co doping on the preferred catalyst activity, we calculated the Tafel slope to analyze the reaction kinetics of the catalyst. In Figure 4c, Ni_1.01_Co_0.99_P NDs/NF exhibit the Tafel slope of 65.4 mV dec^−1^, which is much smaller than that of Ni_1.51_Co_0.49_P NDs/NF (102.5 mV dec^−1^), Ni_1.32_Co_0.68_P NDs/NF (87.6 mV dec^−1^), Ni_0.67_Co_1.33_P NDs/NF (71.3 mV dec^−1^), Ni_0.52_Co_1.48_P NDs/NF (92.4 mV dec^−1^), and Ni_2_P NDs/NF (89.5 mV dec^−1^). This result demonstrates Ni_1.01_Co_0.99_P NDs/NF’s excellent HER reaction kinetics and also reflects the enhanced HER activity of Ni site via the introduction of Co. Additionally, the electron transfer rate can elucidate the kinetics of electrocatalytic reactions. As a consequence, we conducted electrochemical impedance spectroscopy test for further investigation. As shown in Figure 4d, the Nyquist plot was tested at an overpotential of 0.5 V vs. RHE. The smaller semicircle represents a smaller charge transfer resistance and a faster charge transfer rate. EIS shows that the semicircle diameter of Ni_1.01_Co_0.99_P NDs/NF is the smallest, indicating that Ni_1.01_Co_0.99_P NDs/NF (Rct = 3.48 Ω) have the lowest charge transfer obstruction in the electrolyte. To further understand HER performance, the CV method was applied to derive the electrochemical active surface area (ECSA) by analyzing the measurement data of double-layer capacitance (C_dl_) (Figure S4). Ni_1.01_Co_0.99_P NDs/NF also possess the highest C_dl_ value of 19.56 mF∙cm^−2^, which is 3.55, 3.05, 2.51, 2.26, and 16.16 times higher than that of Ni_1.51_Co_0.49_P NDs/NF, Ni_1.32_Co_0.68_P NDs/NF, Ni_0.67_Co_1.33_P NDs/NF, Ni_0.52_Co_1.48_P NDs/NF and Ni_2_P NDs/NF, respectively (Figure S5). The calculated ECSA values of all catalysts are listed in Table S4, and the ECSA of Ni_1.01_Co_0.99_P NDs/NF was also significantly higher than that of other samples. The LSV curve decays slightly after 5000 CV cycles, indicating the excellent stability of Ni_1.01_Co_0.99_P NDs/NF (Figure 4e). In addition, at the overpotential of 136 mV, the initial current density of 100 mA cm^−2^ decays by only 5.9%, and the current density of 500 mA cm^−2^ decays by 4.3% after 30 h of timing potential determination, further confirming the good stability of Ni_1.01_Co_0.99_P NDs/NF (Figure 4f and Figure S6).

**Figure 5:**
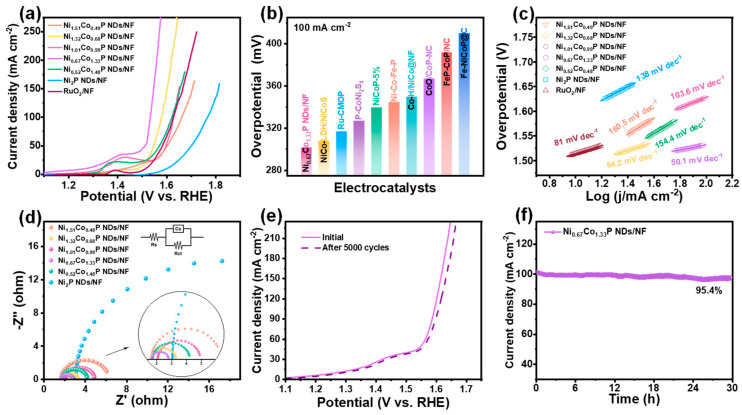
Electrocatalytic performance of Ni_x_Co_2−x_P NDs/NF for OER with different Ni/Co ratios in 1 M KOH: (**a**) iR-corrected LSV profiles; (**b**) overpotential comparison at 100 mA cm^−2^ with recently reported electrocatalysts; (**c**)Tafel plots; (**d**) EIS spectra (inset shows the corresponding equivalent impedance circuit diagram); (**e**) comparison of LSV profiles of Ni_0.67_Co_1.33_P NDs/NF before and after 5000 CV cycle; (**f**) time-dependent current density curve of Ni_0.67_Co_1.33_P NDs/NF under static potential of 0.5 V for 30 h.

The OER performance of prepared electrocatalysts was also measured with the same electrochemical test system as the HER. It can be revealed from Figure 5a and Figure S7 that Ni_0.67_Co_1.33_P NDs/NF demonstrate exceedingly good OER performance. For Ni_0.67_Co_1.33_P NDs/NF, achieving the current density of 100 mA∙cm^−2^ necessitates only a 298 mV overpotential, which is much lower than that of Ni_1.51_Co_0.49_P NDs/NF (431 mV), Ni_1.32_Co_0.68_P NDs/NF (348 mV), Ni_1.01_Co_0.99_P NDs/NF (398 mV), Ni_0.52_Co_1.48_P NDs/NF (391 mV), and Ni_2_P NDs/NF (536 mV). That is even better than that of commercial RuO_2_ (397 mV). This is mainly because Ni/Co bimetallic adjusting reduces the oxygen affinity of the Co atom. Remarkably, in Figure 5b and Table S5, the overpotential at 100 mA cm^−2^ of Ni_0.67_Co_1.33_P NDs/NF is superior to that of most reported TMPs for OER [43,44,45,50,51,52,53,54,55]. As displayed in Figure 5c, the Ni_0.67_Co_1.33_P NDs/NF exhibit the smallest Tafel slope of 50.1 mV dec^−1^, obviously superior to that of RuO_2_ (81 mV∙dec^−1^), Ni_1.51_Co_0.49_P NDs/NF (160.5 mV∙dec^−1^), Ni_1.32_Co_0.68_P NDs/NF (84.2 mV∙dec^−1^), Ni_1.01_Co_0.99_P NDs/NF (103.6 mV∙dec^−1^), Ni_0.52_Co_1.48_P NDs/NF (154.4 mV∙dec^−1^), and Ni_2_P NDs/NF (138 mV∙dec^−1^). In the low-frequency range (Figure 5d), the Nyquist plots of Ni_0.67_Co_1.33_P NDs/NF indicate that the charge–transfer resistance is the lowest when compared with other semicircles. In stability testing, the performance in LSV curves remains unchanged after 5000 CV cycles (Figure 5e). Meanwhile, chronoamperometry testing at a stationary overpotential of 302 mV demonstrates the excellent stability of Ni_0.67_Co_1.33_P NDs/NF with a current density of 100 mA cm^−2^ maintained for 30 h. When reaching 500 mA cm^−2^ at a voltage of 1.32 V, the current density decays by 3.6% after 30 h (Figure 5f and Figure S10).

### 2.3. Overall Water Splitting Electrocatalytic Activity

**Figure 6:**
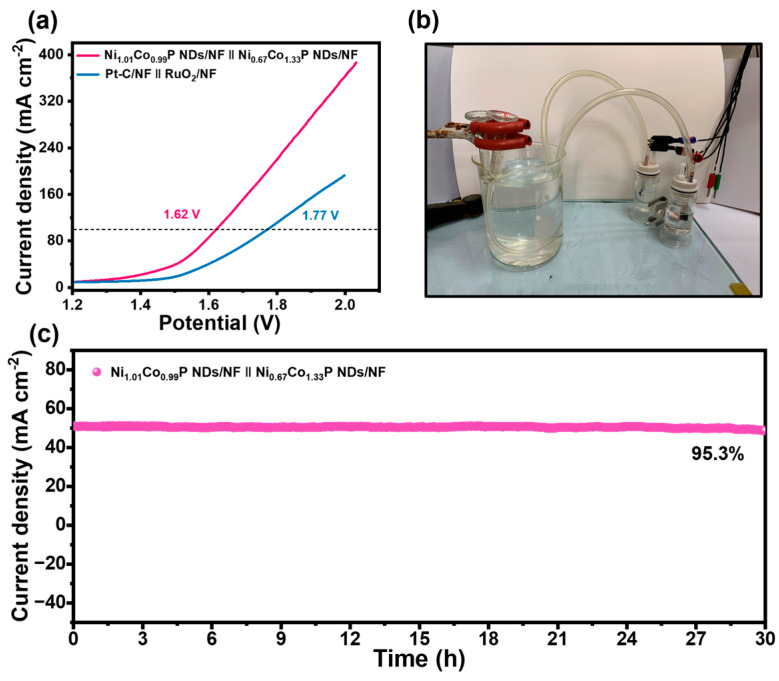
(**a**) Overall water splitting performance of Ni_1.01_Co_0.99_P NDs/NF‖Ni_0.67_Co_1.33_P NDs/NF and Pt-C/NF‖RuO_2_ in 1.0 M KOH; (**b**) photograph of H-type alkaline water electrolytic cell using with Ni_0.67_Co_1.33_P NDs/NF and Ni_1.01_Co_0.99_P NDs/NF as the anode and cathode; (**c**) time-dependent current density curve for Ni_1.01_Co_0.99_P NDs/NF‖Ni_0.67_Co_1.33_P NDs/NF at a potential of 1.58 V.

Given the results above, Ni_1.01_Co_0.99_P NDs/NF and Ni_0.67_Co_1.33_P NDs/NF were applied as competent electrocatalysts for overall water electrolysis. Ni_0.67_Co_1.33_P NDs/NF was employed as an anode, and Ni_1.01_Co_0.99_P NDs/NF as a cathode to assemble a two-electrode alkaline electrolyzer containing 1 M KOH. At the potential values of 1.62 and 1.90 V, current densities could achieve 100 and 150 mA cm^−2^, respectively (Figure 6a). The activity is outstanding compared with that of recent reported electrolyzers fabricated with bifunctional electrocatalysts (Table S7). In addition, the outstanding performance could fulfill the demands (1.8–2.4 V up to 200–400 mA∙cm^−2^) of commercial catalysts for electrolytic water. In Figure 6c and Figure S11, it is evident that there is no noticeable current variation after continuous operation at 1.57 V for 30 h compared with Pt-C/NF‖RuO_2_/NF electrode, which reveals that Ni_1.01_Co_0.99_P NDs/NF‖Ni_0.67_Co_1.33_P NDs/NF electrode has excellent durability. It is worthwhile mentioning that plenty of gas bubbles were generated on the surface of Ni_1.01_Co_0.99_P NDs/NF‖Ni_0.67_Co_1.33_P NDs/NF electrode.

### 2.4. DFT Calculations

**Figure 7:**
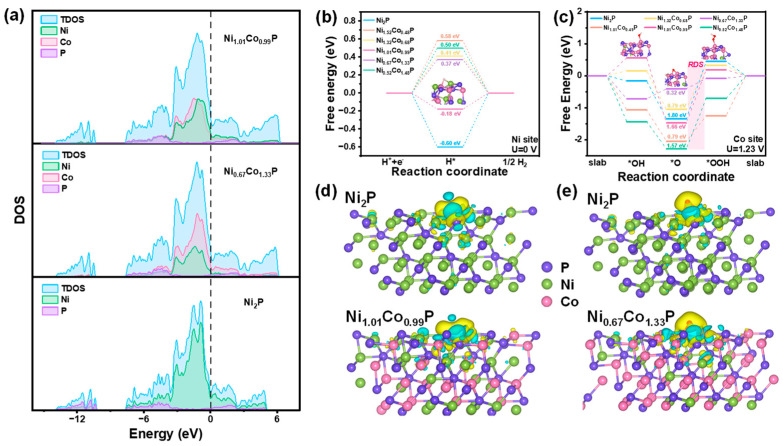
(**a**) Total and partial electronic density of states (TDOS and PDOS) calculated for Ni_1.01_Co_0.99_P, Ni_1.01_Co_0.99_P, and Ni_2_P. Schematic energy profiles of different models for (**b**) HER pathway and (**c**) OER pathway. Calculated charge density differences of (**d**) Ni_2_P and Ni_1.01_Co_0.99_P for HER and (**e**) Ni_2_P and Ni_0.67_Co_1.33_P for OER. The Ni, Co, and P atoms are marked in green, rose red, and purple, respectively. The yellow and cyan regions refer to increased and decreased charge distributions, respectively.

To elucidate the impact of Co-doping on the HER and OER activity, density functional theory (DFT) calculations were performed to elucidate the alterations in reaction energy and electronic structure pre- and post-incorporation of Co. The surfaces are simulated by a slab model with an exposed crystal plane of (111), and the metal atoms of Ni and Co were considered as active sites for HER and OER, respectively. The total densities of states (DOS) of Ni_2_P, Ni_1.01_Co_0.99_P, and Ni_0.67_Co_1.33_P are shown in Figure 7a. It is apparent that the DOS of Ni_2_P, Ni_1.01_Co_0.99_P, and Ni_0.67_Co_1.33_P are continuously observed near Fermi level, which implies that they possess excellent conductivity. Furthermore, the intensities of Ni_1.01_Co_0.99_P and Ni_0.67_Co_1.33_P at Fermi level are stronger than that of Ni_2_P, indicating the bimetal synergistic effect significantly increases the conductivity of Ni_1.01_Co_0.99_P and Ni_0.67_Co_1.33_P, which are profitable for HER and OER, respectively.

For the HER process, the Gibbs free energy of *H is employed as the important indicator in evaluating the HER performance. The overpotential (η) at the Ni site of Ni_2_P, as depicted in Figure 7b, exhibits a value of 1.43 V, which decreases after the introduction of Co., and the η of Ni_1.01_Co_0.99_P (0.19 V) is smaller than that of Ni_1.51_Co_0.49_P, Ni_1.32_Co_0.68_P, Ni_0.67_Co_1.33_P, and Ni_0.52_Co_1.48_P, respectively, indicating that *H is most easily absorbed on Ni_1.01_Co_0.99_P. On the Co site, the ∆G_*H_ value of Ni_1.01_Co_0.99_P remains the smallest. However, in comparison with the Ni site, there is little variation among all samples, suggesting a minor influence of Co on the HER activity (Figure S12). Therefore, Ni is suggested to be the main active site for all the catalysts, and the addition of Co can cooperate with Ni to improve HER performance. For OER processes, a four-step mechanism including all intermediates (*OH, *O, and *OOH) at Ni and Co sites were calculated and recorded as Ni-Ni_x_Co_2−x_P and Co-Ni_x_Co_2−x_P, respectively. As shown in Figure 7c, it is suggested that the rate-determining step (RDS) for the OER is the third step (*O conversion into *OOH), which exhibits the highest ΔG. Compared with the ΔG of RDS in Ni-NiCoP, the ΔG of RDS is only 0.37 eV on Co-Ni_0.67_Co_1.33_P. While the ΔG of RDS in Co-Ni_1.51_Co_0.49_P, Co-Ni_1.01_Co_0.99_P, Co-Ni_1.32_Co_0.68_P, and Co-Ni_0.52_Co_1.48_P are 0.97 eV, 2.03 eV, 1.37 eV, 1.57 eV, respectively (Figure S13). The calculation results show that the incorporated Co becomes the active site of OER and effectively reduces the reaction energy barrier of the RDS in the reaction, thereby enhancing the OER performance of Ni_x_Co_2−x_P.

Charge density difference was calculated on the same sites to evaluate the alteration of the electronic structure after Co doping in Ni_2_P. In Figure 7d,e, the green regions represent the charge consumption, while the yellow refers to the charge accumulation. The charge density difference of Ni_2_P and Ni_1.01_Co_0.99_P on the Ni site and Co site was calculated to explore the charge transfer. The charge accumulation region around the H atom on the Ni site of Ni_1.01_Co_0.99_P is more intensive than that of Ni_2_P and Co-Ni_1.01_Co_0.99_P, and the corresponding charge depletion region shows the trend of consumption (Figure 7d and Figure S14). This phenomenon reveals that Co doping leads to enhanced electron transfer between H and Ni atoms, thus reducing the ΔG_*H_ of the Ni site. Then charge density difference was calculated for *O in Ni_2_P and Ni_0.67_Co_1.33_P (Figure 7e and Figure S11). Compared with Ni-Ni_0.67_Co_1.33_P and Ni-Ni_2_P, O atoms obtained more electrons in Co-Ni_0.67_Co_1.33_P. The charge loss from the Co atom in the Co-Ni_0.67_Co_1.33_P system is more significant than Ni in the Ni-Ni_2_P and Ni-Ni_0.67_Co_1.33_P, indicating that the appropriate doping of Co could make it an effective active site in the OER process, thus decreasing the adsorption energy of O atoms and the overpotential.

## 3. Experimental Section

### 3.1. Materials

None of the materials were further purified: Ni(NO_3_)_2_·6H_2_O (AR, 98%), CO(NO_3_)_2_·6H_2_O (AR, 99%), urea (AR, 99%), and NaH_2_PO_2_·H_2_O (99%) were purchased from Aladdin Reagent. The source of NH_4_F (AR, 99.6%), KOH (AR, 85%), HCl (AR), and ethanol (AR, 99.7%) was Sinopharm Chemical Reagent Co., Ltd, Shanghai, China. NF (99.8%, thickness 1 mm, porosity 98%, PPI 110, aperture 0.2–0.6 mm) was purchased from Sinero Energy.

### 3.2. Materials Preparation

Due to the oxygen in the environment, it was necessary to remove the oxide layer on NF by pretreatment first. A piece of NF ($60\times 10\times 1\;mm^{3}$) was washed using 3 M HCl, ethanol, and DI water by ultrasonicating. Then, the NF was dried in a vacuum oven at 80 °C for 6 h.

For the synthesis of Ni_x_Co_2−x_-pre., a hydrothermal method was employed. In order to adjust the Ni/Co molar ratio, Ni(NO_3_)_2_∙6H_2_O (2.25 mmol, 2 mmol, 1.5 mmol), Co(NO_3_)_2_∙6H_2_O (1.5 mmol, 2 mmol, 2.25 mmol), urea (15 mmol), and NH_4_F (7.5 mmol) were evenly dispersed in 60 mL distilled water, respectively, with continuous stirring for 30 min. The formed homogeneous solution and the NF were placed in a 100 mL autoclave Teflon-lined autoclave reactor and remained at 140 °C for 10 h in a drying oven. After washing with distilled water and ethanol, and drying at 60 °C for 10 h, the Ni_x_Co_2−x_ precursors/NF (Ni_x_Co_2−x_-pre.) were obtained. Then, the prepared precursors and 2 g NaH_2_PO_2_·2H_2_O were placed in two porcelain boats and then heated to 350 °C in the Ar atmosphere at the rate of 2 °C∙min^−1^ maintained for 3 h. It is important to note that NaH_2_PO_2_·2H_2_O was positioned upstream of the tubular furnace. Finally, the Ni_x_Co_2−x_P NDs/NF were manufactured after cooling to ambient temperature, cleansing with DI water and ethanol, and drying at 80 °C for 1 h with a vacuum. According to the calculation, the loading mass of the sample on the foam nickel is about 4 mg/cm^2^.

### 3.3. Material Characterization

X-ray diffraction (XRD, DX2700, Haoyuan Instrument, Dandong, Liaoning, China) was utilized to reflect the crystalline structure between the 2θ range 10° and 80°. The precursor preparation for XRD measurement is as follows: Hydrothermal treatment was used to strip the precursor from NF. The stripped-down precursor powders were ground for 2–3 min in the agate mortar and then sprinkled into the sample slot of the glass sample holder. A cover glass was used to gently press the surface of the sample and scrape off the excess powder. The bimetallic phosphide preparation for the XRD measurement is as follows: The prepared sample was cut into $2\times 2\;cm^{2}$ squares, and a square was fixed onto the sample stage with conductive adhesive for XRD measurement. ICP-5000 was applied for analyzing inductively coupled plasma atomic emission spectroscopy (ICP–AES). Morphology and structure were determined by field emission scanning electron microscopy (FESEM; JSM-7001F, JEOL, Tokyo, Japan; Sigma500, Zeiss, Germany). Transmission electron microscopy (TEM) and high-resolution TEM (HRTEM) were used to observe the microstructure of the catalysts on a JEM-2100 F transmission electron microscope (TEM, JEOL, Tokyo, Japan). X-ray photoelectron spectroscopy (XPS) was used to determine Ni_x_Co_2−x_P NDs/NF surface elemental composition and valence state using a K-Alpha electron spectrometer (Thermo Scientific, Oxford, UK) with monochromatic Al Kα radiation (1486.6 eV 12 kV).

### 3.4. Electrochemical Measurements

Catalyst electrochemical measurements were conducted at room temperature with the application of the CHI760E electrochemical workstation in a three-electrode system. The NF (1 cm × 1 cm) supported with catalysts was applied as working electrodes, while the Ag/AgCl electrode and graphite rod (for HER) or platinum foil (for OER) were used as the reference electrode and the counter electrode, respectively. For comparison, 8.0 mg of 20 wt% Pt/C or RuO_2_ was dispersed in a mixture of ethanol (150 μL), deionized water (150 μL), and Nafion (5 wt%, 30 μL). After that, 110 μL of catalyst ink was painted into 1 cm × 1 cm NF (loading 4 mg/cm^2^). The undermentioned formula resulted in the conversion of the measured potential into the relative value of the reversible hydrogen electrode (RHE):

$$E\left(RHE\right)=E_{Ag/AgCl}+0.197V+0.059\times pH$$

Before the test, cyclic voltammetry (CV) was used to test 100 cycles until the curves were stable. The measurements of linear scan voltammetry (LSV) were put into effect with the scan rate of 1 mV·s^−1^ in 1M KOH, and the HER and OER curves performed with 90% iR compensation. When testing the electrochemical impedance spectroscopy (EIS) with an amplitude of 5 mV, the frequency was set at the range from 10 kHz to 0.01 Hz. The double-layer capacitance (C_dl_) was estimated by CV through various sweep rates (20, 40, 60, 80, 100, 120 mV∙s^−1^). The ECSA can be calculated based on the formula $ECSA=C_{dl}/C_{S}$ [54], where the value of the specific capacitance (C_S_) is 0.040 mF cm^−2^; the value of the C_dl_ is shown in Tables S4 and S6. The efficiency of water splitting was evaluated in an electrolytic cell, which is a two-electrode system with Ni_1.01_Co_0.99_P NDs/NF as the anode and Ni_0.67_Co_1.33_P NDs/NF as the cathode. The chronoamperometry method (i-t curves) was used to test the durability at a stationary overpotential. In addition, the two-electrode tests were carried out without iR compensation.

## 4. Conclusions

In this work, a series of overall water electrocatalysts (Ni_x_Co_2−x_P NDs/NF) with high catalytic performance were successfully obtained with varied Ni/Co bimetallic ratios. XPS and DFT calculation results reveal that the synergistic effect of bimetal can change the charge distribution, thereby optimizing the electronic structure of the active sites, and greatly reducing the ΔG of RDS. Ni_1.01_Co_0.99_P NDs/NF‖Ni_0.67_Co_1.33_P NDs/NF electrolyzer shows eminent catalytic performance for overall water splitting in 1 M KOH. This work will provide an effective way and theoretical direction to design a stable and effective electrocatalytic decomposition of water.

## Data Availability

Data are contained within the article and supplementary materials.

## Supplementary Materials

The following supporting information can be downloaded at: https://www.mdpi.com/article/10.3390/molecules29030657/s1. Figure S1: XRD patterns of Ni_x_Co_2−x_-pre. powders; Figure S2: XPS full spectra of (a) Ni_1.01_Co_0.99_P NDs/NF and (b) Ni_0.67_Co_1.33_P NDs/NF; Figure S3: The effects of phosphating on the HER activity of the Ni_1.01_Co_0.99_P NDs/NF in 1 M KOH electrolyte; Figure S4: CV curves at different scanning rates 20, 40, 60, 80, 100, 120 mV s^−1^ for HER in 1.0 M KOH; Figure S5: C_dl_ values of different catalysts for HER in 1.0 M KOH; Figure S6: Time-dependent current density curve of Ni_1.01_Co_0.99_P NDs/NF under static potential of −1.24 V for 30 h; Figure S7: The effects of phosphating on the OER activity of the Ni_0.67_Co_1.33_P NDs/NF in 1 M KOH electrolyte; Figure S8: CV curves at different scanning rates of 20, 40, 60, 80, 100, 120 mV s^−1^ for OER in 1.0 M KOH; Figure S9: C_dl_ values of different catalysts for OER in 1.0 M KOH; Figure S10: Time-dependent current density curve of Ni_0.67_Co_1.33_P NDs/NF under static potential of 1.32 V for 30 h; Figure S11: Time-dependent current density curve for Pt-C/NF‖RuO_2_/NF at a potential of 1.58 V; Figure S12: Reaction free-energy diagrams for HER of Co site on Ni_x_Co_2−x_P; Figure S13: Reaction free-energy diagrams for OER of Ni site on Ni_x_Co_2−x_P at 0 V; Figure S14: Calculated charge density differences of (a) Co-Ni_1.01_Co_0.99_P for HER and (b) Ni-Ni_0.67_Co_1.33_P for OER; Table S1: ICP-AES data and atomic ratio of Ni/Co in Ni_x_Co_2−x_P NDs/NF on Ni foam.; Table S2: The binding energy and percent of each species in XPS spectra; Table S3: Comparison of HER performance with other reported electrocatalysts; Table S4: The value of ECSA for each catalyst investigated in 1 M KOH for HER.; Table S5: Comparison of OER performance with other reported electrocatalysts; Table S6. The value of ECSA for each catalyst investigated in 1 M KOH for OER; Table S7: Comparison of overall water splitting performance with other reported electrocatalysts. References [34,37,38,41,42,43,44,45,48,49,52,55,56,57,58,59,60] are cited in the supplementary materials.
