# Supplementary material for: Electronic Structure Regulated Nickel-Cobalt Bimetal Phosphide Nanoneedles for Efficient Overall Water Splitting
Source: Molecules. 2024 Jan 31;29(3):657. doi: 10.3390/molecules29030657 (PMC10856751; doi:10.3390/molecules29030657)
Supplement: Supplementary file 1 [file molecules-29-00657-s001.zip › molecules-2826080-supplementary.pdf]

Supporting Information

Table S1. ICP-AES data and atomic ratio of Ni/Co in $Ni_xCo_{2-x}P$ NDs/NF on Ni foam.

Samples	Feeding ratios	m_{Ni} (mg)	m_{Co} (mg)	Atomic ratios
$Ni_{1.51}Co_{0.49}P$	3/1	2.974	0.971	1/0.326
$Ni_{1.32}Co_{0.68}P$	2/1	3.327	1.715	1/0.515
$Ni_{1.01}Co_{0.99}P$	1/1	3.456	3.363	1/0.973
$N_{0.67}Co_{1.33}P$	1/2	0.811	1.619	1/1.996
$Ni_{0.51}Co_{1.49}P$	1/3	0.956	2.720	1/2.845

Table S2 The binding energy and percent of each species in XPS spectra.

Sample	Binding	Bonding Energy (eV)	Percentage (%)
**Ni_2P NDs/NF**	The satellite peaks of Ni; (Co); (P) 2p	861,879.7; 0,0; 129.8,130.5	59.8; 0; 5.2
	Oxidized Ni; (Co) species	855.7,873.4; 0,0	37.3; 0
	Ni-P; (Co-P); (P-O)	852.6,870.3; 0,0; 134	2.9; 0; 94.8
**$Ni_{1.51}Co_{0.49}P$ NDs/NF**	The satellite peaks of Ni; (Co); (P) 2p	861,879.3; 786.3,803.2; 129.5,130.1	68.7; 53.5; 6.3
	Oxidized Ni; (Co) species	855.7,873.5; 781.4,797.1	28.3; 32.7
	Ni-P; (Co-P); (P-O)	853,867.6; 778.3,793.4; 134.9	3; 13.8; 93.7
**$Ni_{1.32}Co_{0.68}P$ NDs/NF**	The satellite peaks of Ni; (Co); (P) 2p	862.1,880.6; 786.2,803.3; 129.3,130	56.8; 62.6; 2.8
	Oxidized Ni; (Co) species	856.7,874.2; 782,798.1	41.4; 33.2
	Ni-P; (Co-P); (P-O)	852.7,868.4; 777.7,794.1; 134.7	1.8; 4.2; 97.2
**$Ni_{1.01}Co_{0.99}P$ NDs/NF**	The satellite peaks of Ni; (Co); (P) 2p	861.7,880.6; 785.3,803; 129.2,129.7	57.8; 59.9; 5.5
	Oxidized Ni; (Co) species	856.8,874.8; 781.7,797.9	40; 28.6
	Ni-P; (Co-P); (P-O)	853.2,869.5; 778.3,793.4; 133.5	2.2; 11.5; 94.5
**$Ni_{0.67}Co_{1.33}P$ NDs/NF**	The satellite peaks of Ni; (Co); (P) 2p	861.3,880.4; 785.9,803.3; 129.2,130.1	59.7; 61.9; 2.9
	Oxidized Ni; (Co) species	856.9,874.8; 781.9,797.8	34.3; 33.8
	Ni-P; (Co-P); (P-O)	853.1,870.5; 778.5,793.2; 133.9	6; 4.3; 97.1
**$Ni_{0.52}Co_{1.48}P$ NDs/NF**	The satellite peaks of Ni; (Co); (P) 2p	862,880.7; 786.1,802.8; 129.4,130.2	63.8; 51.7; 3.7
	Oxidized Ni; (Co); species	856.7,874.5; 782,798.2	33.7; 31.8
	Ni-P; (Co-P); (P-O)	852.8,870.9; 778.2,792.3; 134.5	2.5; 16.5; 96.3

Table S3. Comparison of HER performance with other reported electrocatalysts.

Catalysts	iR correction	Substrate	η_{10} (mV)	Tafel slope (mV/dec)	Ref.
**$Ni_{1.01}Co_{0.99}P$ NDs/NF**	90%	NF	66	65.4	This work
**Ni5%CoP**	100%		84	67	46
**NiCoP-5%**	-		88	41	44
**CoP@NiCoP**	-	CC	71	148	38
**0.4-Co_2P/Ni_xP_y@NF**	90%	NF	80	86	34
**Mo-NiCoP-3**	100%		76	60	42
**FeP-CoP/NC**	Without iR correction	NC	79	71	43
**Co-NixPy@Co_3O_4**	100%	NF	72	56.26	41
**G@Co-W-P**	100%		91.5	61.2	48
**NiFe-LDH/NiCoP**	Without iR correction	NF	120	88.2	49
**P-$CoNi_2S_4$**	100%		135	65	45

Table S4. The value of ECSA for each catalyst investigated in 1 M KOH for HER.

Catalysts	C_{dl}/mF cm^{-2}	ECSA/cm^2
**$Ni_{1.01}Co_{0.99}P$ NDs/NF**	19.56	489
**Ni_2P NDs/NF**	1.21	30.25
**$Ni_{1.51}Co_{0.49}P$ NDs/NF**	5.5	137.5
**$Ni_{1.32}Co_{0.68}P$ NDs/NF**	6.42	160.5
**$Ni_{0.67}Co_{1.33}P$ NDs/NF**	7.8	195
**$Ni_{0.52}Co_{1.48}P$ NDs/NF**	8.65	216.25

Table S5. Comparison of OER performance with other reported electrocatalysts.

Catalysts	iR correction	Substrate	η_{100} (mV)	Tafel slope (mV/dec)	Ref.
**$Ni_{0.67}Co_{1.33}P$ NDs/NF**	90%	NF	298	50.1	This work
**NiCoP-5%**	-		~330	66	44
**NiCoP/NF**	100%	NF	~310	49.5	57
**0.4-Co_2P/Ni_xP_y@NF**	90%	NF	300	162	34
**Mo-NiCoP**	90%	NF	~320	49.4	56
**Fe-NiCoP@C**	Without iR correction	NF	410	36	55
**Ni-Co-Fe-P**	-		~340	85.64	52
**Co_2P/Ni_2P-2%Mo**	Without iR correction	NF	357	46.1	57
**FeP-CoP/NC**	Without iR correction	NC	392	73	43
**CoO-CoP**	90%	NF	365	90	54

Table S6. The value of ECSA for each catalyst investigated in 1 M KOH for OER.

Catalysts	C_{dl}/mF cm^{-2}	ECSA/cm^2
**$Ni_{0.67}Co_{1.33}P$ NDs/NF**	17.46	436.5
**Ni_2P NDs/NF**	0.65	16.25
**$Ni_{1.51}Co_{0.49}P$ NDs/NF**	11.3	282.5
**$Ni_{1.32}Co_{0.68}P$ NDs/NF**	6	150
**$Ni_{1.01}Co_{0.99}P$ NDs/NF**	3.24	81
**$Ni_{0.52}Co_{1.48}P$ NDs/NF**	1.02	25.5

Table S7. Comparison of Overall water splitting performance with other reported electrocatalysts.

Catalysts	Substrate	Cell voltage (V) at 100 mA cm^{-2}	Ref.
**$Ni_{0.67}Co_{1.33}P$ NDs/NF ‖ $Ni_{0.67}Co_{1.33}P$ NDs/NF**	NF	1.62	This work
**0.4-Co_2P/Ni_xP_y@NF ‖ 0.4-Co_2P/Ni_xP_y@NF**	NF	1.74	44
**NiCoP/CC ‖ NiCoP/CC**	NF	~1.77	37
**CeO_2-NiCoP ‖ CeO_2-NiCoP**	NF	~1.68	34
**Mo-NiCoP ‖ Mo-NiCoP**	NF	1.70	56
**NiCoP/NF ‖ NiCoP/NF**	NF	1.74	57
**NFP500-30 ‖ NFP500-30**	NF	1.76	58

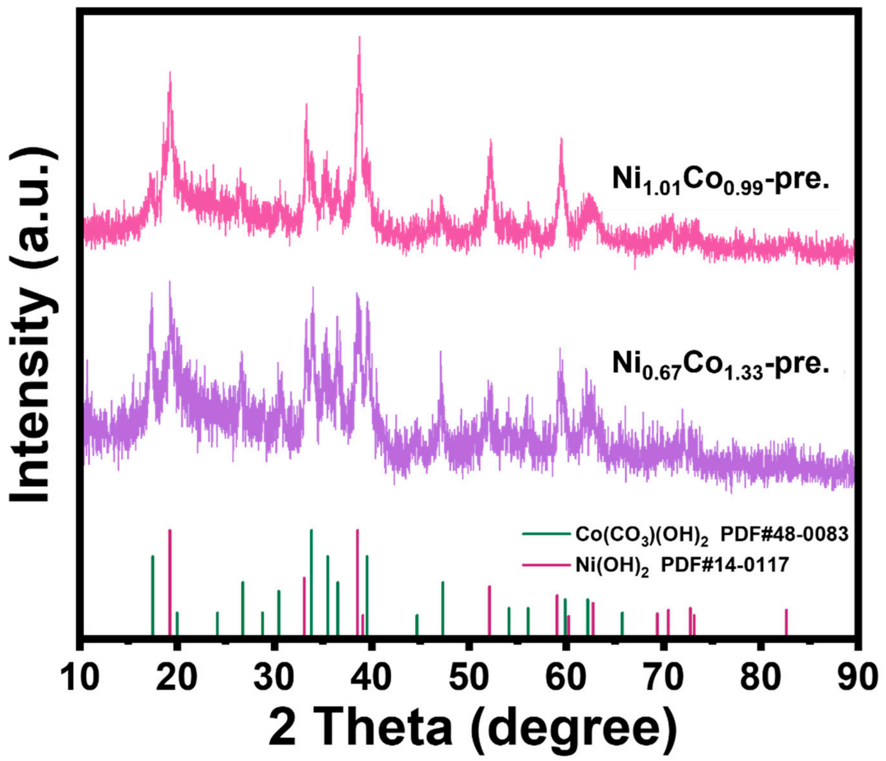

Figure S1. XRD patterns of Ni_xCo_{2-x}-pre. without NF.

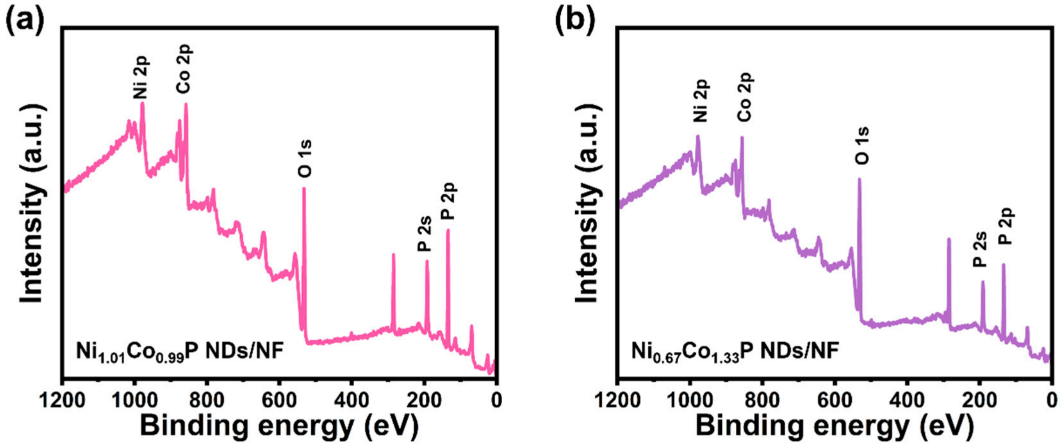

Figure S2. XPS full spectra of (a) $Ni_{1.01}Co_{0.99}P$ NDs/NF and (b) $Ni_{0.67}Co_{1.33}P$ NDs/NF.

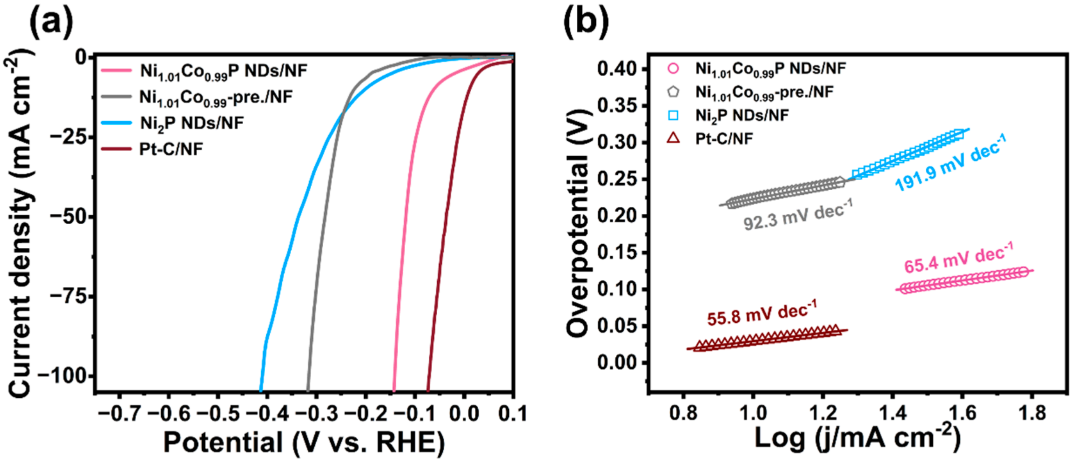

Figure S3. The effects of phosphating on the HER activity of the $Ni_{1.01}Co_{0.99}P$ NDs/NF in 1 M KOH electrolyte. (a) LSV curves for HER, (b) The Tafel plots corresponding to HER LSV curves

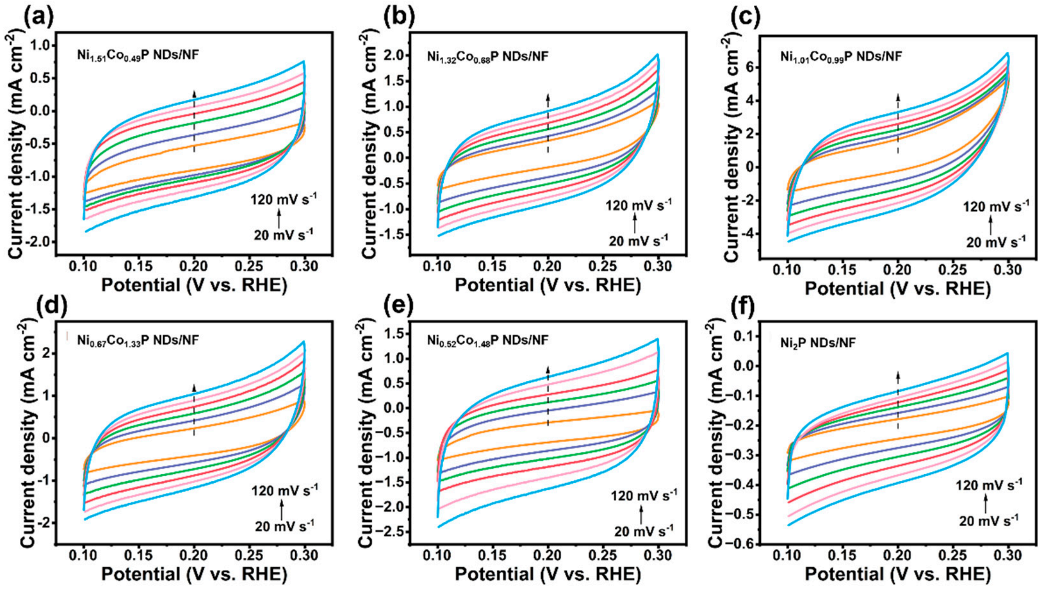

Figure S4. CV curves at different scanning rates 20, 40, 60, 80, 100, 120 mV s^{-1} for HER in 1.0 M KOH

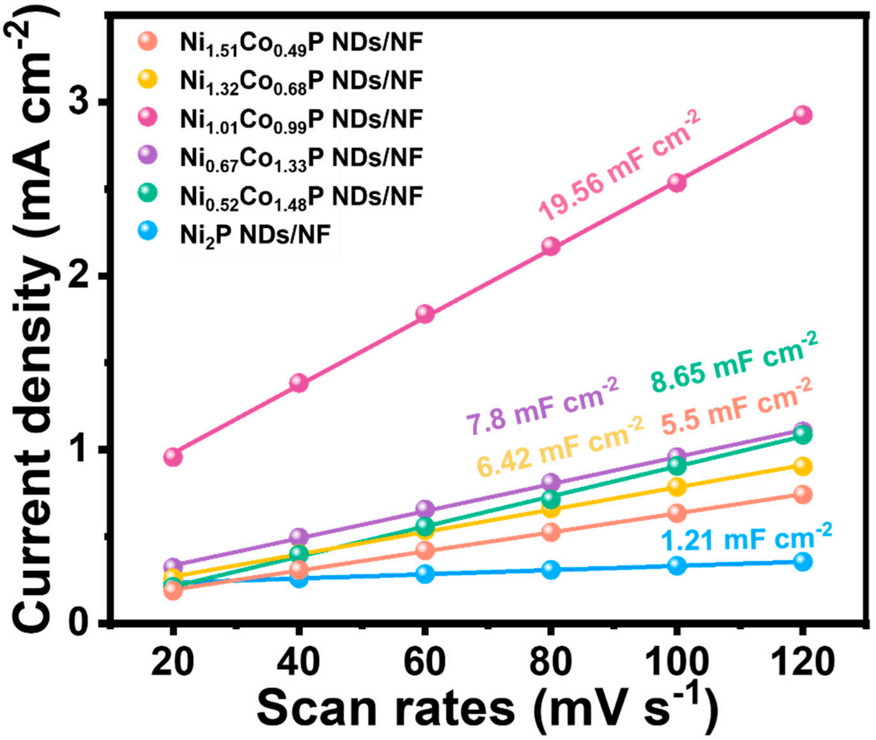

Figure S5. Cdl values of different catalysts for HER in 1.0 M KOH

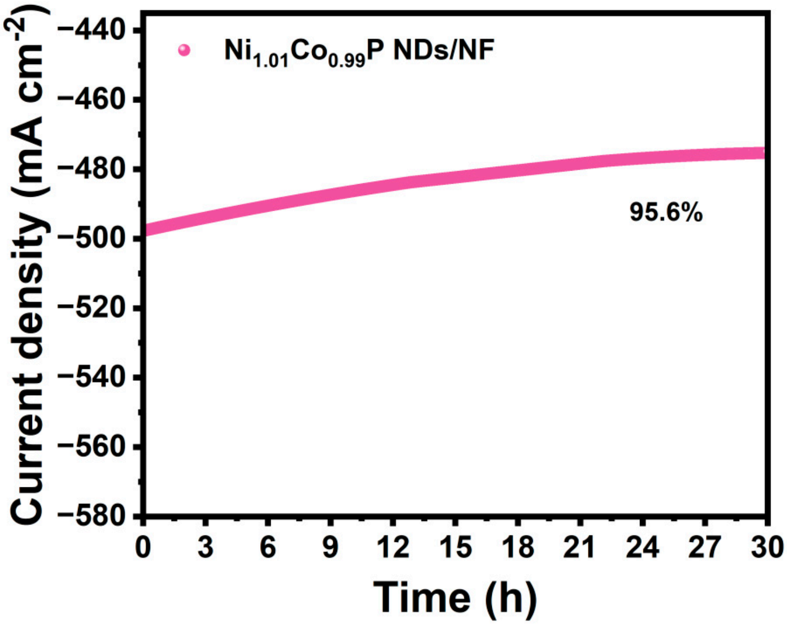

Figure S6. Time-dependent current density curve of $Ni_{1.01}Co_{0.99}P$ NDs/NF under static potential of -1.24 V for 30 h.

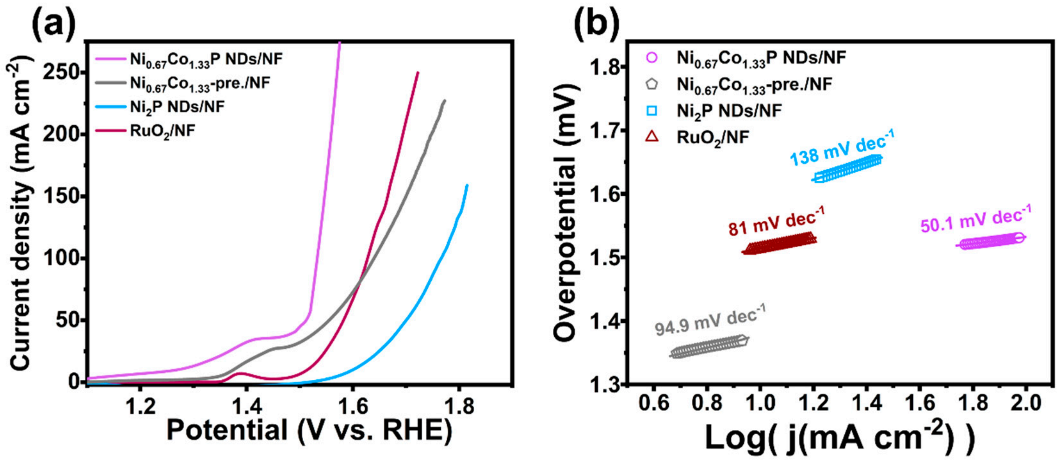

Figure S7. The effects of phosphating on the OER activity of the $Ni_{0.67}Co_{1.33}P$ NDs/NF in 1 M KOH electrolyte. (a) LSV curves for OER, (b) The Tafel plots corresponding to HER LSV curves.

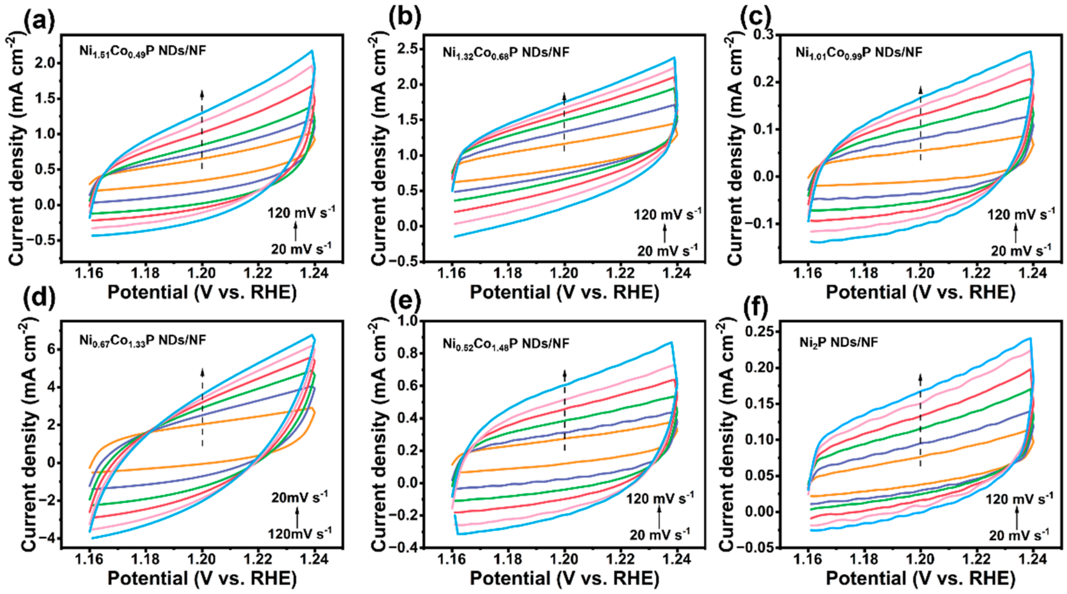

Figure S8. CV curves at different scanning rates of 20, 40, 60, 80, 100, 120 mV s^{-1} for OER in 1.0 M KOH

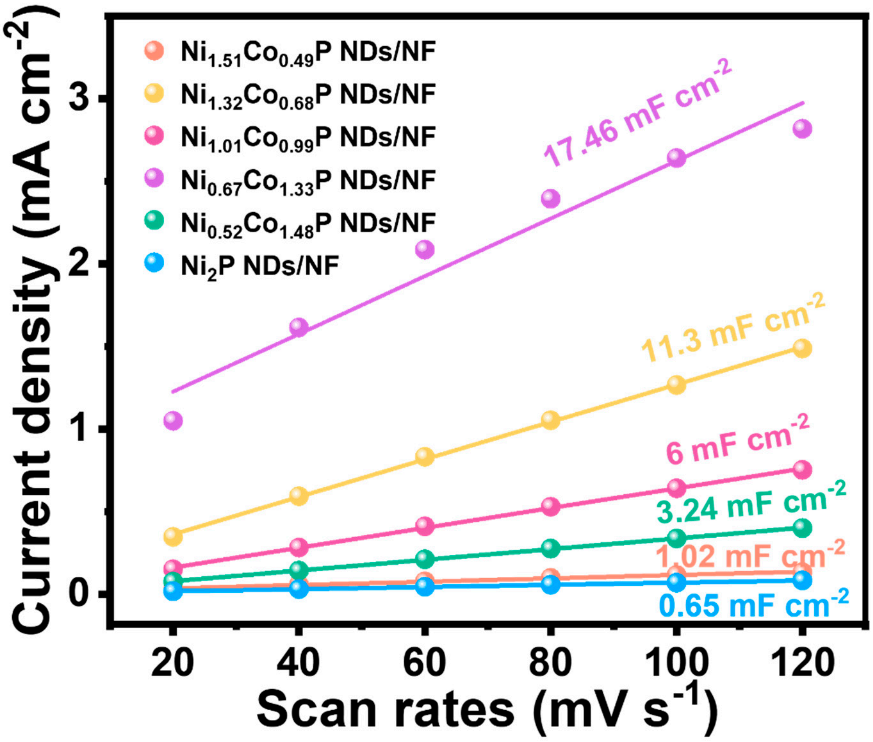

Figure S9. Cdl values of different catalysts for OER in 1.0 M KOH

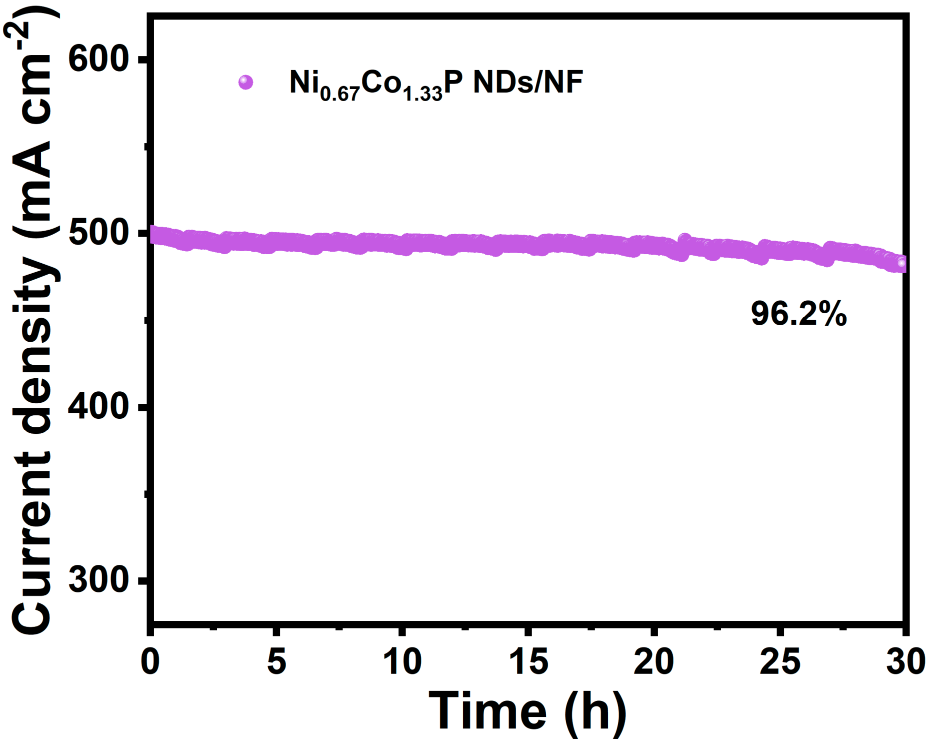

Figure S10. Time-dependent current density curve of $Ni_{0.67}Co_{1.33}P$ NDs/NF under static potential of 1.32 V for 30 h.

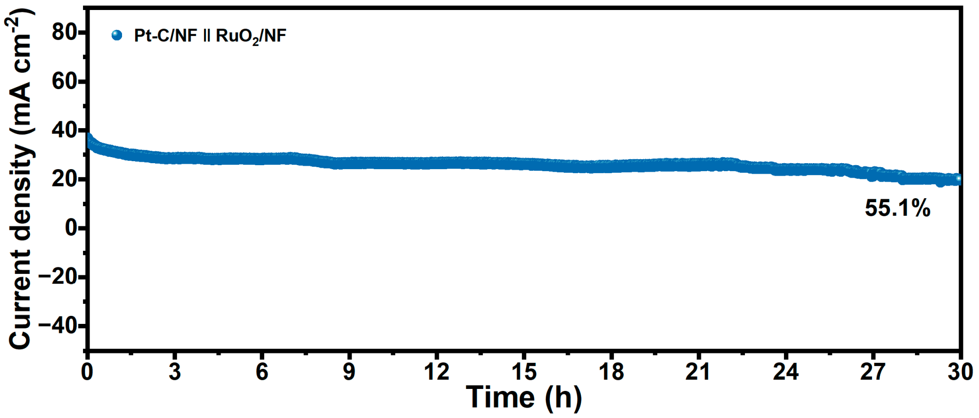

Figure S11. Time-dependent current density curve for Pt-C/NF ‖ RuO_2/NF at a potential of 1.58 V

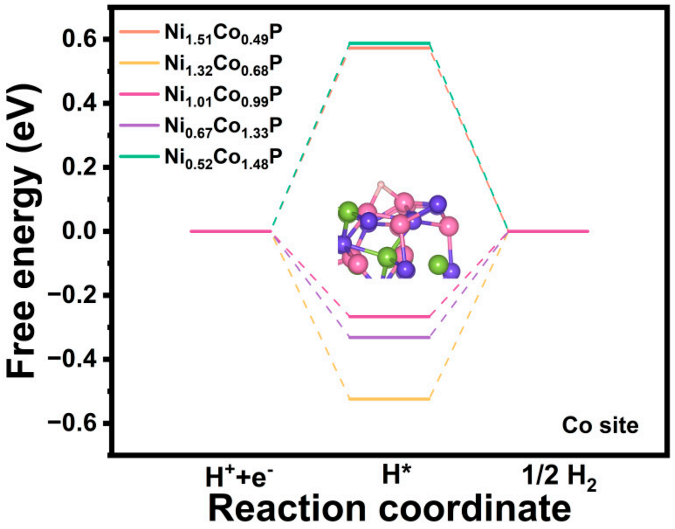

Figure S12. Reaction free-energy diagrams for HER of Co site on $Ni_xCo_{2-x}P$.

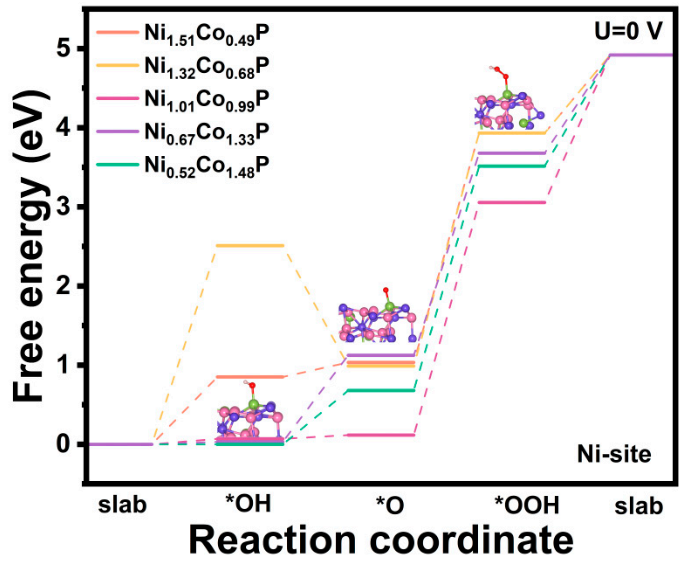

Figure S13. Reaction free-energy diagrams for OER of Ni site on $Ni_xCo_{2-x}P$ at 0 V.

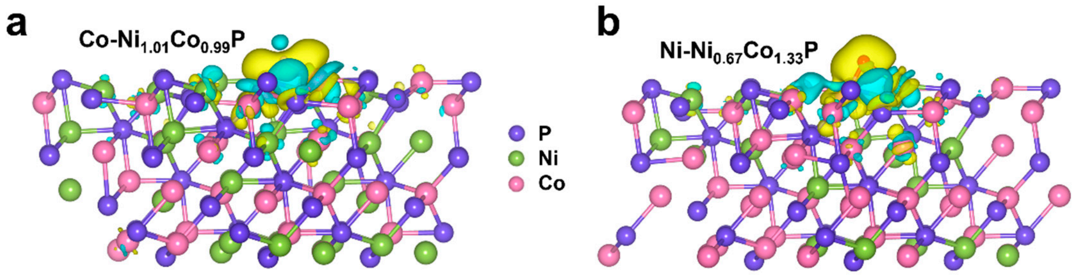

Figure S14. Calculated charge density differences of (a) $Co-Ni_{1.01}Co_{0.99}P$ for HER and (b) $Ni-Ni_{0.67}Co_{1.33}P$ for OER. The Ni, Co, and P atoms are marked in green, rose red, and purple, respectively. The yellow and cyan regions refer to increased and decreased charge distributions, respectively.

References

[34] H. Liu, Y. Zhang, R. Ge, J.M. Cairney, R. Zheng, A. Khan, S. Li, B. Liu, L. Dai, W. Li, Tailoring the electronic structure of Ni_5P_4/Ni_2P catalyst by Co_2P for efficient overall water electrolysis, Appl. Energy 349 (2023), 121582.

[37] C. Du, L. Yang, F. Yang, G. Cheng, W. Luo, Nest-like NiCoP for Highly Efficient Overall Water Splitting, ACS Catal. 7(6) (2017) 4131-4137.

[38] R. Hu, L. Jiao, H. Liang, Z. Feng, B. Gao, X.F. Wang, X.Z. Song, L.Z. Liu, Z. Tan, Engineering Interfacial Built-in Electric Field in Polymetallic Phosphide Heterostructures for Superior Supercapacitors and Electrocatalytic Hydrogen Evolution, Small (2023), 2304132.

[41] B. Lu, J. Zang, W. Li, J. Li, Q. Zou, Y. Zhou, Y. Wang, Co-doped Ni_xP_y loading on Co_3O_4 embedded in Ni foam as a hierarchically porous self-supported electrode for overall water splitting, Chem. Eng. J. 422 (2021), 130062.

[42] J. Lin, Y. Yan, C. Li, X. Si, H. Wang, J. Qi, J. Cao, Z. Zhong, W. Fei, J. Feng, Bifunctional Electrocatalysts Based on Mo-Doped NiCoP Nanosheet Arrays for Overall Water Splitting, Nano-Micro Letters 11 (2019), 55.

[43] X. Yan, J. Biemolt, K. Zhao, Y. Zhao, X. Cao, Y. Yang, X. Wu, G. Rothenberg, N. Yan, A membrane-free flow electrolyzer operating at high current density using earth-abundant catalysts for water splitting, Nat. Commun. 12 (2021) 1-9.

[44] Y. Zhao, J. Zhang, Y. Xie, B. Sun, J. Jiang, W. Jiang, S. Xi, H.Y. Yang, K. Yan, S. Wang, X. Guo, P. Li, Z. Han, X. Lu, H. Liu, G. Wang, Constructing Atomic Heterometallic Sites in Ultrathin Nickel-Incorporated Cobalt Phosphide Nanosheets

via a Boron-Assisted Strategy for Highly Efficient Water Splitting, Nano Letters 21 (2021) 823-832.

[45] X.F. Lu, S.L. Zhang, W.L. Sim, S. Gao, X.W. Lou, Phosphorized $CoNi_2S_4$ Yolk-Shell Spheres for Highly Efficient Hydrogen Production via Water and Urea Electrolysis, Angew. Chem. Int. Ed. 60 (2021) 22885-22891.

[46] L. Zhao, M. Wen, Y. Tian, Q. Wu, Y. Fu, A novel structure of quasi-monolayered NiCo-bimetal-phosphide for superior electrochemical performance, J. Energy Chem. 74 (2022) 203-211.

[48] X. Wang, J.B. Le, Y. Fei, R. Gao, M. Jing, W. Yuan, C.M. Li, Self-assembled ultrasmall mixed Co–W phosphide nanoparticles on pristine graphene with remarkable synergistic effects as highly efficient electrocatalysts for hydrogen evolution, J. Mater. Chem. A. 10 (2022) 7694-7704.

[49] H. Zhang, X. Li, A. Hähnel, V. Naumann, C. Lin, S. Azimi, S.L. Schweizer, A.W. Maijenburg, R.B. Wehrspohn, Bifunctional Heterostructure Assembly of NiFe LDH Nanosheets on NiCoP Nanowires for Highly Efficient and Stable Overall Water Splitting, Adv. Funct. Mater. 28 (2018), 1706847.

[52] Y. Wang, Y. Wang, H. Gao, Z. Huang, Q. Hao, B. Liu, Interface-induced contraction of core–shell Prussian blue analogues toward hollow Ni-Co-Fe phosphide nanoboxes for efficient oxygen evolution electrocatalysis, Chem. Eng. J. 451 (2023), 138515.

[54] K. Chen, Y. Cao, W. Wang, J. Diao, J. Park, V. Dao, G.-C. Kim, Y. Qu, I.-H. Lee, Effectively enhanced activity for overall water splitting through interfacially strong

P–Co-O tetrahedral coupling interaction on CoO/CoP heterostructure hollow-nanoneedles, J. Mater. Chem. A 11 (2023) 3136-3147.

[55] Y. Kang, S. Wang, S. Zhu, H. Gao, K.S. Hui, C.-Z. Yuan, H. Yin, F. Bin, X.-L. Wu, W. Mai, L. Zhu, M. Hu, F. Liang, F. Chen, K.N. Hui, Iron-modulated nickel cobalt phosphide embedded in carbon to boost power density of hybrid sodium–air battery, Appl. Catal. B Environ. 285 (2021), 119786.

[56] Y. Zhao, J. Chen, S. Zhao, W. Zhou, R. Dai, X. Zhao, Z. Chen, T. Sun, H. Zhang, A. Chen, Mo-doped NiCoP nanowire array grown in situ on Ni foam as a high-performance bifunctional electrocatalyst for overall water splitting, J. Alloys Compd. 918 (2022), 165802.

[57] H. Liu, M. Jin, D. Zhan, J. Wang, X. Cai, Y. Qiu, L. Lai, Stacking faults triggered strain engineering of ZIF-67 derived Ni-Co bimetal phosphide for enhanced overall water splitting, Appl. Catal. B Environ. 272 (2020), 118951.

[58] H.-S. Hu, Y. Li, Y.-R. Shao, K.-X. Li, G. Deng, C.-B. Wang, Y.-Y. Feng, NiCoP nanorod arrays as high-performance bifunctional electrocatalyst for overall water splitting at high current densities, J. Power Sources 484 (2021), 229269.

[59] R. Narasimman, G. Jnanapriya, S. Sujatha, S.A. Ilangovan, Vertically aligned nickel-iron-phosphide nanosheets on three-dimensional porous nickel foam for overall water splitting, J. Alloys Compd. 947 (2023), 169474.
